# Improved Bowel Preparation with Multimedia Education in a Predominantly African-American Population: A Randomized Study

**DOI:** 10.1155/2016/2072401

**Published:** 2016-02-23

**Authors:** Shashank Garg, Mohit Girotra, Lakshya Chandra, Vipin Verma, Sumanjit Kaur, Allawy Allawy, Alessandra Secco, Rohit Anand, Sudhir K. Dutta

**Affiliations:** ^1^Division of Gastroenterology, Department of Medicine, Sinai Hospital of Baltimore, Baltimore, MD 21215, USA; ^2^Johns Hopkins University-Sinai Program in Internal Medicine, Baltimore, MD 21215, USA; ^3^Department of Medicine, University of Maryland School of Medicine, Baltimore, MD 21215, USA

## Abstract

*Background and Aim*. Inadequate bowel preparation is a major impediment in colonoscopy quality outcomes. Aim of this study was to evaluate the role of multimedia education (MME) in improving bowel preparation quality and adenoma detection rate.* Methods*. This was an IRB-approved prospective randomized study that enrolled 111 adult patients undergoing outpatient screening or surveillance colonoscopy. After receiving standard colonoscopy instructions, the patients were randomized into MME group (*n* = 48) and control group (*n* = 46). The MME group received comprehensive multimedia education including an audio-visual program, a visual aid, and a brochure. Demographics, quality of bowel preparation, and colonoscopy findings were recorded.* Results*. MME group had a significantly better bowel preparation in the entire colon (OR 2.65, 95% CI 1.16–6.09) and on the right side of the colon (OR 2.74, 95% CI 1.12–6.71) as compared to control group (*p* < 0.05). Large polyps (>1 cm) were found more frequently in the MME group (11/31, 35.5% versus 0/13; *p* < 0.05). More polyps and adenomas were detected in MME group (57 versus 39 and 31 versus 13, resp.) but the difference failed to reach statistical significance.* Conclusion*. MME can lead to significant improvement in the quality of bowel preparation and large adenoma detection in a predominantly African-American population.

## 1. Introduction

Colon cancer is the second most common cancer and also the second leading cause of cancer-related mortality in the United States (http://www.cancer.org/acs/groups/content/@editorial/documents/document/acspc-044552.pdf). Colonoscopy has become the investigation of choice for colorectal cancer (CRC) screening [1]. Several studies have shown that CRC screening with colonoscopy reduced the colon cancer incidence and mortality [2–8]. Quality of bowel preparation is one of the major factors that determine the detection of adenomas during the screening colonoscopy and also the interval to the next colonoscopic examination [1, 9, 10]. However, there are limited data on the effect of patient education in improving the quality of bowel preparation prior to screening colonoscopy [11, 12]. The aim of this study was to evaluate the effect of comprehensive multimedia education (MME) in improving quality of bowel preparation and adenoma detection rate in a predominantly African-American population presenting to an inner city hospital for screening or surveillance colonoscopy.

## 2. Materials and Methods

### 2.1. Study Design

This was an IRB-approved prospective, randomized single blind study conducted in the Division of Gastroenterology at Sinai Hospital of Baltimore between September 2012 and December 2013. All authors had access to the study data and had reviewed and approved the final paper.

### 2.2. Inclusion Criteria

All adult patients (>18 years of age) undergoing screening or surveillance colonoscopy performed by a single gastroenterologist at Sinai Hospital of Baltimore during the study period were included.

### 2.3. Exclusion Criteria

Patients unable to provide informed consent, patients admitted to the hospital, patients with a known colorectal cancer, or patients undergoing colonoscopy for a reason other than screening or surveillance for colon polyps were excluded.

### 2.4. Randomization

After obtaining informed consent, all of the study patients received standard colonoscopy preparation instructions by the participating gastroenterologist as follows:

*Standard Instructions*

1. Take only clear liquid diet on the day before the colonoscopy. Avoid any red or purple color fluids to prevent confusion with blood in the colon.
2. On the evening prior to colonoscopy, start taking the Golytely^©^ bowel preparation at 6 p.m. Take 8 ounces every 20 minutes and finish by midnight.

A research associate then randomized the patients using simple randomization into study or the multimedia education (MME) group and control group. The gastroenterologist was blinded to the patient group assignment.

### 2.5. Multimedia Education

MME group patients received multimedia education by 3 methods from the research assistant in an education room in the gastroenterology clinic as follows:

*Multimedia Education*

**Figure 1:**
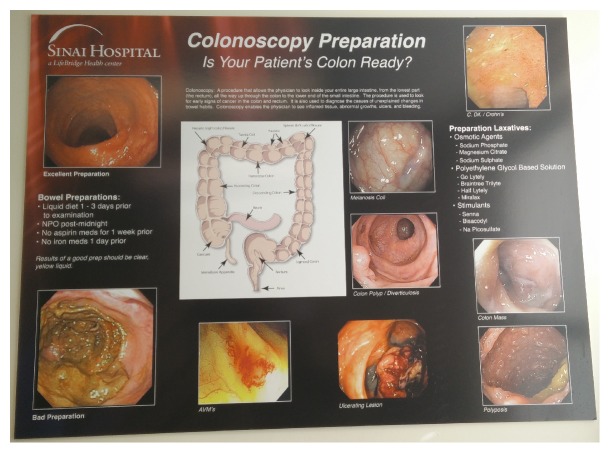
Visual aid.

1. Visual aid: Figure 1.
2. Audio-visual education: Supplemental file 1 in Supplementary Material available online at http://dx.doi.org/10.1155/2016/2072401.
3. American College of Gastroenterology (ACG) questions and answers about quality in colonoscopy: Appendix* Link*: http://patients.gi.org/gi-health-and-disease/questions-and-answers-about-quality-in-colonoscopy/.

First, they were shown a visual aid by the research assistant. It included pictures of good and poor bowel preparation and types of polyps and cancers seen in the colon (Figure 1). Second, patients were given audio-visual education with 3 videos with a total time of 13 minutes and 52 seconds (supplemental file 1). Issues addressed with audio-visual program included importance of CRC prevention by screening or surveillance colonoscopy, importance of good bowel preparation towards doing a good colonoscopic examination, and patient expectations from the bowel preparation. Third, they were given a brochure by American College of Gastroenterology addressing the importance of bowel preparation (see Appendix). After the MME session, the patients were encouraged to seek clarification about bowel preparation instructions from the research assistant.

### 2.6. Data Collection

Patient demographics, past medical history, results of prior colonoscopy(s), and family history of colon cancer were recorded. After the procedure, cecal intubation and scope withdrawal times, quality of bowel preparation, number of polyps, size, location, morphology (sessile or pedunculated), and pathology of the polyps were recorded.

### 2.7. Assessing the Quality of Bowel Preparation

After each procedure, the participating gastroenterologist assessed the quality of bowel preparation based on modified Aronchick scale (Table 1). Aronchick scale is a validated scale used to assess the quality of bowel preparation based on percentage of colon wall visualized [13]. The scale was modified to separately assess the preparation of the right and the left colon. Right side of the colon was defined as the portion of colon extending from cecum to distal transverse colon. Left side of the colon was defined portion of colon extending from splenic flexure to rectum. The category of <90% bowel wall visualized in Aronchick scale was further subcategorized into 2 categories (75–89% and <75%) for a more objective measurement of the bowel preparation quality (Table 1).

### 2.8. End Points

Primary end points of the study were difference in the quality of bowel preparation and and polyp and adenoma detection rate between the MME group and the control group. Secondary end points included difference in the quality of bowel preparation and polyp and adenoma detection rate separately in the right and left side of the colon between the 2 groups. Post hoc analysis was done for adenoma size and morphology in the 2 groups.

### 2.9. Data Analysis

Categorical variables were analyzed using chi-square (*n* > 5) or Fischer's exact test (*n* ≤ 5). Continuous variables were analyzed using *t*-test or Wilcoxon rank-sum test depending on distribution. Ordinal data were analyzed using ordinal regression. The data obtained from the patients were analyzed according to their initial assigned group. A two-sided *p* value < 0.05 was considered as significant. The data was analyzed using STATA (Version 13.1, ^©^StataCorp, TX, USA).

## 3. Results

**Figure 2:**
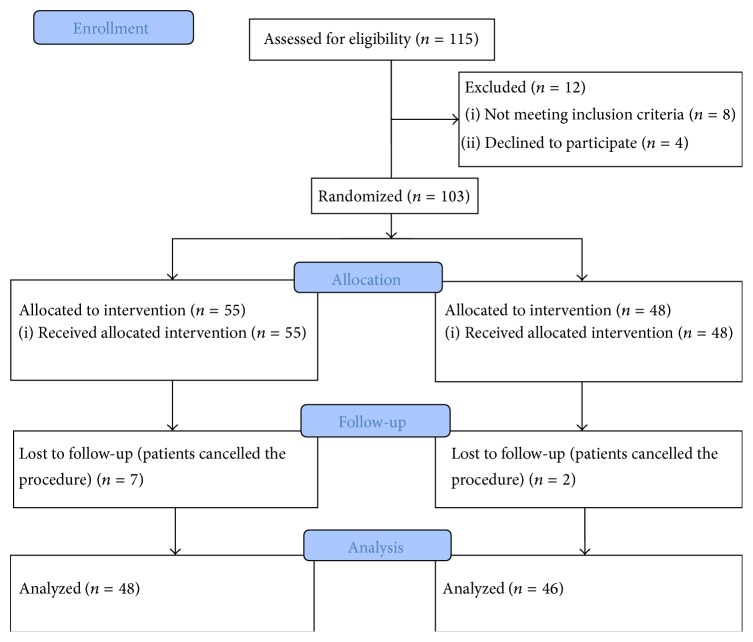


A total of 115 patients underwent screening or surveillance colonoscopy by the participating gastroenterologist during the study period. Four patients declined to participate in the study. Eight patients were excluded after initial enrolment as per exclusion criteria defined above (colon cancer *n* = 3, right hemicolectomy for tubulovillous adenoma with high grade dysplasia *n* = 1, rectal bleeding *n* = 3, and chronic diarrhea *n* = 1). A total of 107 patients were randomized into 2 groups, 55 cases and 48 controls. Seven patients from the study group and 2 patients from control group missed the appointment and did not undergo the procedure. A total of 94 patients (48 cases and 46 controls) were included in the final analysis (Figure 2).

Mean age was 59.27 ± 18.08 years for study group and 57.28 ± 19.40 years for control group (*p* > 0.05). Gender and race distribution in study and control group were similar (*p* > 0.05, Table 2). The two groups were also similar in terms of number of patients with prior colonoscopy, history of colon polyps, and family history of colon cancer.

Cecal intubation times were similar in MME and control group (median time of 6 minutes and range of 2–27 minutes versus median time of 6 minutes and range of 2–15 minutes; *p* = NS). Similarly scope withdrawal times were similar in both groups (median time of 12 minutes and range of 6–45 minutes versus median time of 13 minutes and range of 6–41 minutes; *p* = NS). The quality of bowel preparation in the entire colon was significantly better in the MME group as compared to control group (OR 2.65, 95% CI 1.16–6.09, *p* < 0.05, Table 3). Similarly, quality of bowel preparation on the right side of the colon was significantly better in the MME group (OR 2.74, 95% CI 1.12–6.71, *p* < 0.05, Table 4). This difference in the quality of bowel preparation in the entire colon and on the right side of the colon between the two groups remained significant when controlled for age, gender, race, and previous colonoscopy. Quality of bowel preparation in the left side of colon was comparable in the 2 groups (*p* > 0.05).

A higher number of polyps and adenomas were detected in the MME group (57 and 31, resp.) as compared to control group (39 and 13, resp.). Similarly, number of adenomas detected on the right side of the colon was higher in the MME group (19 versus 8). However, these differences failed to reach statistical significance (*p* > 0.05). Similarly, polyp and adenoma detection rate were similar between the MME group (47.91% and 33.33%, resp.) and the control group (34.78% and 19.56, resp.; *p* > 0.05). When classified by size, large polyps were found more commonly in the MME group (11/31, 35.48%) compared to control group (0/13; *p* < 0.05; Table 4).

## 4. Discussion

This study successfully demonstrates a significant improvement in the quality of bowel preparation in the entire colon and particularly in the right side of the colon with the use of comprehensive multimedia education in a predominantly African-American population. Our results are consistent with previously reported studies on patient education in improving quality of bowel preparation in the general population in US [12, 14–16] and other countries [11, 17, 18].

Our study employed 3 different methods of patient education. First, the patients were educated, using the visual aide, about the difference between a good and poor bowel preparation and how a good bowel preparation can help in detecting polyps and masses in the colon. Second, audio-visual program was conducted under direct supervision. Third, the patients were provided with a brochure approved by a professional society to read. In contrast, previous studies have utilized only 1 method of patient education, that is, phone based [15], online video based [14], visual aid [17], or reading material based [12] education. Moreover, the patient education was not directly supervised directly in the GI clinic in most of these studies. Instead, the patients were provided with the educational material to read or view at home. This is the first study that was able to show the efficacy of MME in increasing the detection of large polyps in the study group. This finding could be related to the provision of supervised education to the patients by trained health care providers.

Quality of bowel preparation is vital to performing effective screening or surveillance examination of the colon. It has been shown that adenoma miss rates range within 20–41% from index colonoscopy at 1–3 years when quality of bowel preparation is suboptimal [9, 19]. Poor understanding of the process of bowel preparation by patients remains a major barrier in achieving high rates of good quality bowel preparation at a population level [20–22]. This factor seems to be particularly relevant to African-American population as supported by the fact that African-Americans have a high rate of interval cancer and significantly higher mortality from colorectal cancer compared to other races and ethnic groups (http://www.cdc.gov/cancer/colorectal/statistics/race.htm). Appannagari et al. reported that African-Americans had the highest rate of suboptimal bowel preparation quality (43% versus 25.5% in Caucasians, *p* < 0.001) in large retrospective study involving 3741 subjects [23]. Moreover, poor understanding of the process of bowel preparation has been shown to contribute to inadequate bowel preparation in this population [24].

MME can address the issues related to importance of bowel preparation and CRC screening at individual and population level especially in African-Americans. The audio-visual component of such education supplements the information given to the patient during physician-patient interaction. It provides an opportunity for patients to understand the appearance of different types of polyps and the importance of a good bowel preparation in detecting these polyps. It can also address difficulties of doing a bowel preparation and may help patients deal with these issues with a scientific approach. MME sessions can be performed for a group of patients by trained health care providers utilizing such educational aides.

The strengths of this study include the prospective randomized, single blind design and performance of all the procedures by a single gastroenterologist that avoids interobserver bias. The limitation of the study includes small sample size.

In conclusion, this study showed a significant improvement in the quality of bowel preparation in African-American population undergoing screening and surveillance colonoscopies. These observations need to be further examined in a large multicenter prospective randomized study.

## Supplementary Material

Video used for audio-visual education of the patients in the multimedia education (MME) group. The first part of the video provides information about the importance of screening or surveillance colonoscopy and provides a detail of what to expect on the day of the procedure. The second part of the video addresses the importance of bowel preparation and provides different solutions for doing a good bowel preparation at home. The third video addresses the potential harmful effects of a poor bowel preparation including increased risk of missing polyps during the colonoscopy.

## Figures and Tables

**Table 1:** Representation of bowel preparation assessment with modified Aronchick scale.

Score	% bowel wall visualized	Right colon	Left colon	Entire colon
1	≥90			

2	75–89			

3	<75			

**Table 2:** Demographics and clinical characteristics of the study population.

	Study group (*n* = 48)	Control group (=46)	*p* value
Mean age in years (±2SD)	59.27 ± 18.08	57.28 ± 19.40	0.31
Gender (F : M)	27 : 21	25 : 21	0.85
Race (AA : other)	43 : 5	41 : 5	0.94
Prior colonoscopy	17	16	0.95
History of colon polyps	4	2	0.68
Family history of colon cancer	8	4	0.36

**Table 3:** Colonoscopy parameters and quality of bowel preparation in the study population.

	Study group (*n* = 48)	Control group (=46)	*p* value
Median cecal intubation time in minutes (range)	6 (2–27)	6 (2–15)	0.85
Median scope withdrawal time in minutes (range)	12 (6–45)	13 (6–41)	0.13
Quality of bowel preparation in the entire colon			
(1) >90%	34	22	0.02
(2) 75–90%	11	17	
(3) <75%	3	7	
Quality of bowel preparation in the right colon			
(1) >90%	38	26	0.03
(2) 75–90%	7	16	
(3) <75%	3	4	
Quality of bowel preparation in the left colon			
(1) >90%	37	28	0.09
(2) 75–90%	9	14	
(3) <75%	2	4	

**Table 4:** Clinical and histological features of colonic polyps detected in the study population.

	Study group (*n* = 48)	Control group (*n* = 46)	*p* value
Total number of adenomas/carcinomas detected	31	13	0.1
(1) Right sided	19	8	
(2) Left sided	12	5	
Total number of small adenomas	20	13	0.38
Total number of large adenomas/carcinomas	11	0	0.03
Total number of sessile adenomas	27	13	0.11
Total number of pedunculated adenomas	2	0	0.33
Adenoma detection rate (%)	16/48 (33.33%)	9/46 (19.56%)	0.13
(1) Right sided	12	7	
(2) Left sided	7	3	
Number of polyps detected	57	39	0.21
(1) Right sided	20	11	
(2) Left sided	37	28	
Polyp detection rate (%)	23/48 (47.91%)	16/46 (34.78%)	0.2

## Appendix

### American College of Gastroenterology (ACG) Questions and Answers about Quality in Colonoscopy

*(1) Why Is Quality Important in Colonoscopy?* Although colonoscopy has been available in clinical practice for more than 40 years, only in the past 15 years has awareness developed that the success of colonoscopy in preventing colorectal cancer and minimizing complications is very dependent on the skill and competence of the colonoscopist. Colonoscopists differ substantially in the number of precancerous polyps they detect during colonoscopy and in how often they perform colonoscopy in response to both normal and abnormal findings. Awareness of these differences led the U.S. Multisociety Task Force on Colorectal Cancer in 2002, as well as a joint task force of experts from the American College of Gastroenterology and American Society of Gastrointestinal Endoscopy in 2006, to propose quality indicators that colonoscopists can use to measure how effectively and safely they perform colonoscopy. Obviously, patients have an interest in undergoing the most effective and safe colonoscopy possible, and achieving these goals requires a colonoscopist who is committed to high quality.

*(2) Does the Quality of Examination Differ among Colonoscopists from Different Specialties?* Studies have shown average performance of colonoscopy by gastroenterologists to be superior to that of primary care physicians in three different areas of colonoscopy performance. First, three population-based studies have found that gastroenterologists performing colonoscopy are less likely to miss colorectal cancer than primary care physicians who perform colonoscopy. This may reflect the more extensive training that gastroenterologists receive in this procedure and their higher volumes of colonoscopy in practice. Second, gastroenterologists' patients are less likely to incur serious complications during colonoscopy, such as perforation or making a hole in the colon, compared to primary care physicians. Third, gastroenterologists are less likely than both primary care physicians and general surgeons to perform colonoscopy at intervals that are considered too short according to current guidelines. Whether this difference reflects a lack of confidence among primary care physicians and general surgeons in the quality of their colonoscopy or lack of awareness of current guidelines is unknown.

*(3) Is There Variation in Quality of Performance among Members of the Same Specialty?* Even though gastroenterologists have on average the highest level of training and their examinations have been shown on average to be superior to primary care physicians, there is considerable variation among gastroenterologists in their detection rates of precancerous polyps. Therefore, it is essential that every colonoscopist, regardless of specialty, makes measurements to establish that their examinations are effective. It is very reasonable and appropriate for patients to ask questions of their colonoscopist about whether quality measurements are being made and their results.

*(4) How Can I Be Sure That I Will Receive a Careful Examination of My Colon?* The measurement that best reflects how carefully colonoscopy is performed is a doctor's “adenoma detection rate.” This rate is defined as the percentage of patients aged 50 and older undergoing screening colonoscopy, who have one or more precancerous polyps detected. This rate should be at least 25% in men and 15% in women. A secondary measure of careful examination is that doctors should have an average withdrawal time of at least six minutes. The withdrawal time is the time it takes to remove the scope from the colon. This interval is important because this is the phase of colonoscopy when most doctors actually examine the colon systematically for polyps. It is perfectly reasonable to expect doctors to have measured their adenoma detection rate and to record their withdrawal time. It is also reasonable to ask for a copy of the colonoscopy report that documents that the colonoscope was advanced to the very beginning of the colon and that the landmarks of that portion of the colon (called the “cecum”) have been documented by notation in the report and by photography.

*(5) Why Is Bowel Preparation for Colonoscopy Important, and What Can I Do to Make Sure My Colon Is Thoroughly Cleansed for the Procedure?* Colonoscopy is a video examination of the colon. The video camera and the colonoscope, like any other video camera, cannot see through solids. Therefore, the colon must be thoroughly cleansed to provide the doctor the best opportunity possible for a thorough and detailed examination.

Be sure to pick up and read your written bowel preparation instructions at least several days before your colonoscopy. Go over the instructions and make sure you have all of the materials needed to complete the preparation.

The most effective bowel preparations involve “split” dosing of the laxatives, in which half of the preparation is taken on the morning of the examination, usually 4 to 5 hours before the time of the scheduled colonoscopy, and completed at least 2 to 3 hours before that time. If you are scheduled at 7 or 8 in the morning, this will mean getting up very early to take the second half of the preparation. If the instructions call for split dosing, do not alter the timing of the doses. It is worth the inconvenience of getting up in the middle of the night to make sure that you have a very effective preparation. The timing of the second dose in relationship to the colonoscopy is critical. If too long an interval is allowed between the end of the second half of the preparation and the timing of the colonoscopy, mucus and secretions will come out of the small intestine and stick to the cecum and right colon.

*Summary*. To ensure an effective and safe colonoscopic examination, find a well-trained colonoscopist who is committed to making quality measurements. It is fair to ask the colonoscopist to be sure to do a slow and careful examination and to provide a copy of the report that documents and photographs the complete extent of examination. Take the bowel preparation instructions seriously. Pick up the written instructions early, read them early, and follow them carefully. When colonoscopy is done carefully and with an effective preparation, it is a very powerful cancer prevention technique.
